# Exploring the possible therapeutic role of influenza vaccine in chronic kidney disease patients

**DOI:** 10.1097/MS9.0000000000000357

**Published:** 2023-03-27

**Authors:** Abhigan B. Shrestha, Yasmine A. Mohammed, Tungki P. Umar, Sajina Shrestha, Aashna Mehta, Vikash Jaiswal

**Affiliations:** a M Abdur Rahim Medical College, Dinajpur, Bangladesh; b Faculty of Medicine, Sriwijaya University, Palembang Indonesia; c KIST Medical College, Imadol, Patan, Nepal; d Faculty of Medicine, University of Debrecen, Debrecen, Hungary; e Larkin Community Hospital, South Miami, Florida, USA

**Keywords:** chronic kidney disease, influenza, vaccination

## Abstract

Chronic kidney disease (CKD) is an irreversible change in kidney function and structure with a prevalence of about 9.1% worldwide. Toxins and heavy metal exposure, as well as hypertension and diabetes mellitus, are common causes of CKD. Despite extensive therapeutic options such as renal replacement therapy and renal transplants, most changes in kidney function remain irreversible, causing lifelong morbidity and affecting the quality of life of patients. Increased susceptibility to infections as well as serious complications from influenza, is a major cause of concern in nephrological care. Therefore, it is imperative to consider the protective role of influenza vaccination against seasonal influenza, which can worsen preexisting kidney dysfunction. This commentary explores a possible relationship between the influenza vaccine and patient outcomes in CKD in terms of complications, hospitalization, and possibly prognostic improvements in patient outcomes from CKD.

## Introduction

Chronic kidney disease (CKD) is a noncommunicable disease with high morbidity and mortality as long as the offending factors are not controlled, such as uncontrolled diabetes mellitus, uncontrolled hypertension, elevated proteinuria, and even influenza infection^1^. CKD hampers the immune system and hence reduces the ability of the body to fight against infection. People with CKD are seen to face great difficulties with flu complications, increasing the rate of hospitalization and even death^2^. Hence, the plethora of existing literature regarding the advantages of flu vaccination in CKD patients will be addressed in this article.

Seasonal influenza infection is usually a self-limited disease with no residual complication, but in susceptible populations, it could lead to serious organ damage as acute kidney injury or hasten the course of CKD. It was found that the mortality rate related to a respiratory infection is 10-fold higher in patients with end-stage renal disease compared to the public population^3^. To decrease that mortality risk, the Kidney Disease: Improving Global Outcomes (KDIGO) and the Centers for Disease Control and Prevention (CDC) recommend the seasonal influenza vaccine for CKD patients^4^,^5^. The exact protective effect of vaccination on the renal system is unknown, but it may be explained by preventing kidney injury induced by influenza as acute tubular necrosis or glomerular microthrombi induced by disseminated intravascular coagulation^6^.

Seasonal influenza vaccinations available in the market are mainly of two types: high-dose influenza vaccine (HDV) and standard-dose influenza vaccine (SDV). The HDV contains a higher antigen dose than the SDV. In a randomized controlled trial by Falsey *et al*.^7^, they stated that HDV was associated with a significant increase in antibody levels compared to SDV without a significant increase in relevant adverse events, which suggests that HDV has a higher protective effect. So, the CDC highly recommends the use of HDV in the older population^5^. A recent cohort study performed in Taiwan on patients with type 2 diabetes mellitus found that influenza vaccination decreased the hazard ratio (HR) of CKD/dialysis to 0.47/0.47, 0.48/0.49, and 0.48/0.48 for each influenza season, non-influenza season, and all seasons, respectively^1^; other studies found that vaccination decreases the risk of hospitalization in CKD patients^8^–^10^. A more effective response is found with the next dose of the vaccine, including the booster dose.

The influenza vaccine has not only shown a protective role against influenza but has also shown many other beneficial roles. It has been shown to lower ICU admissions, deep vein thrombosis, and emergency department visits in coronavirus disease 2019 patients compared with those who are unvaccinated^11^. In addition to securing against severe manifestations, influenza vaccination was linked to a decreased probability of severe acute respiratory syndrome coronavirus 2 infection (adjusted odds ratio: 0.86, 95% CI: 0.81–0.91). A meta-analysis has indicated another benefit regarding the cardioprotective effect of the influenza vaccine. This vaccination can reduce the risk of major adverse cardiovascular events, all-cause mortality, cardiovascular mortality, and myocardial infarction^12^.

Because of its cardioprotective properties, influenza vaccination may significantly benefit CKD patients as well. There is a reduced likelihood of hospitalization for the acute coronary syndrome in CKD patients without preexisting cardiovascular disease history who receive influenza vaccination [adjusted hazard ratio (aHR): 0.35] regardless of age category, gender, or periodicity of influenza. The positive impact was correlated with an increase in immunization frequency^13^. Another research discovered that influenza vaccination might reduce the risk of incident peripheral arterial disease in individuals with early-stage CKD but not those with end-stage renal disease^14^. However, the potential effect of the influenza vaccine in CKD patients receiving hemodialysis is promising, with evidence of lesser hospitalization rate (HR: 0.81), cardiovascular disease (HR: 0.85), pneumonia and influenza (HR: 0.80), ICU entry (aHR: 0.20), and death (aHR: 0.50)^9^. Similar effects were noticed in people treated with peritoneal dialysis, where those who had received the influenza vaccine had a lower rate of hospitalization, septicemia, heart disease, ICU management, peritonitis, and mortality than those who had not received the vaccine^15^. Influenza-vaccinated groups also had a lower risk of lung cancer, which appeared to be dose-dependent (lowest aHR: 0.25)^16^. Another recent paper has shown that influenza vaccination reduces the risk of liver cancer^16^.

In conclusion, studies have shown different beneficial roles of the influenza vaccine on CKD patients, like the cardioprotective role, reducing the incidence of peripheral artery disease, hospitalization rate, septicemia, peritonitis, or even cancers. Still, many areas of this field have to be explored and warrant further in-depth higher research. It is highly advisable to take the influenza vaccine as early as a diagnosis is confirmed due to its benefits and lack of many adverse effects. More effective benefits are seen with further doses, including the booster dose.

## Ethical approval

NA.

## Consent

NA.

## Sources of funding

The authors have no funding to declare.

## Author contribution

Substantial contribution to the conception and design of the work: all authors under the guidance of A.B.S. and Y.A.M.; drafting the work and critical revision: all authors under the guidance of A.B.S., A.M., and V.J. All the authors read and approved the final version of the manuscript.

## Conflicts of interest disclosure

The authors have no conflicts of interest to declare.

## Research registration unique identifying number (UIN)

1. Name of the registry: NA.
2. Unique identifying number or registration ID: NA.
3. Hyperlink to your specific registration (must be publicly accessible and will be checked): NA.

## Guarantor

Vikash Jaiswal, Larkin Community Hospital, South Miami, Florida, USA, vikash29jaxy@gmail.com

## Data availability statement

No data are available.

## Provenance and peer review

Not commissioned, externally peer-reviewed.
